# Effect of HIV infection on growth and bone density in peripubertal children in the era of antiretroviral therapy: a cross-sectional study in Zimbabwe

**DOI:** 10.1016/S2352-4642(21)00133-4

**Published:** 2021-08

**Authors:** Ruramayi Rukuni, Andrea M Rehman, Cynthia Mukwasi-Kahari, Tafadzwa Madanhire, Farirayi Kowo-Nyakoko, Grace McHugh, Suzanne Filteau, Joseph Chipanga, Victoria Simms, Hilda Mujuru, Kate A Ward, Rashida A Ferrand, Celia L Gregson

**Affiliations:** a Clinical Research Department, Faculty of Infectious and Tropical Diseases, London School of Hygiene & Tropical Medicine, London, UK; b Biomedical Research and Training Institute, Harare, Zimbabwe; c MRC International Statistics and Epidemiology Group, Department of Infectious Disease Epidemiology, Faculty of Epidemiology and Population Health, London School of Hygiene & Tropical Medicine, London, UK; d Department of Infectious Disease Epidemiology, Faculty of Epidemiology and Population Health, London School of Hygiene & Tropical Medicine, London, UK; e Department of Population Health, Faculty of Epidemiology and Population Health, London School of Hygiene & Tropical Medicine, London, UK; f Department of Radiology, College of Health Sciences, University of Zimbabwe, Harare, Zimbabwe; g MRC Lifecourse Epidemiology Unit, University of Southampton, Southampton, UK; h Department of Paediatrics, University of Zimbabwe, Harare, Zimbabwe; i Musculoskeletal Research Unit, Translational Health Sciences, Bristol Medical School, University of Bristol, Bristol, UK

## Abstract

**Background:**

Faltered linear growth and pubertal delay, which are both common in children with HIV in sub-Saharan Africa, might affect adolescent bone accrual and future fragility fracture risk. We investigated the association of HIV with bone density adjusted for skeletal size in peripubertal children in Zimbabwe.

**Methods:**

We did a cross-sectional study of baseline data from the IMVASK cohort, which enrolled children aged 8–16 years with HIV who had been taking antiretroviral therapy (ART) for at least 2 years, and children of the same age without HIV. Children with HIV were recruited from public sector HIV clinics at Parirenyatwa General Hospital and Harare Central Hospital (Harare, Zimbabwe), and children without HIV were recruited from six schools in the same suburbs that the hospitals serve. Sociodemographic, clinical, and anthropometric data were collected. Dual-energy X-ray absorptiometry (DXA) was used to measure the bone outcomes of total-body less-head bone mineral content for lean mass adjusted for height (TBLH-BMC^LBM^), and lumbar spine bone mineral apparent density (LS-BMAD), and we assessed the prevalence of low TBLH-BMC^LBM^ and low LS-BMAD (defined by Z-scores of less than −2·0). Size adjustment techniques were used to overcome the size dependence of DXA measurement. We used linear regression models, with multiple imputation for missing data, to assess relationships between risk factors and TBLH-BMC^LBM^ and LS-BMAD Z-scores in children with and without HIV.

**Findings:**

We recruited 303 children with HIV (mean age 12·4 years [SD 2·5]; 151 [50%] girls) and 306 children without HIV (mean age 12·5 years [SD 2·5]; 155 [51%] girls). In children with HIV, median age of HIV diagnosis was 3·0 years (IQR 1·2–5·8), and median ART duration was 8·1 years (6·2–9·5); for 102 (34%) children, ART included tenofovir disoproxil fumarate (TDF). Children with HIV had a higher prevalence of low TBLH-BMC^LBM^ Z-score than children without HIV (29 [10%] of 279 children with available data *vs* 18 [6%] of 292 with available data; p=0·066) and a higher prevalence of low LS-BMAD Z-score (40 [14%] of 279 *vs* 17 [6%] of 293 with available data; p=0·0007). HIV and male sex were associated with earlier pubertal (Tanner) stage. The negative associations between HIV and Z-scores for TBLH-BMC^LBM^ and LS-BMAD were more pronounced with pubertal maturation, particularly in girls. Among children with HIV, TDF exposure and orphanhood were associated with lower TBLH-BMC^LBM^ Z-score in confounder-adjusted analysis. Current TDF use (*vs* non-TDF-based ART) was associated with a reduction in TBLH-BMC^LBM^ Z-score of 0·41 (95% CI 0·08–0·74; p=0·015) and in LS-BMAD Z-score of 0·31 (0·08–0·69; p=0·12).

**Interpretation:**

Despite ART, HIV is associated with substantial skeletal deficits towards the end of puberty. The extent of bone deficits associated with TDF and its widespread use in children in sub-Saharan Africa are a concern for future adult fracture risk.

**Funding:**

Wellcome Trust.

## Introduction

Stunting and delayed puberty are common manifestations of perinatally acquired HIV infection; in low-resource settings up to 50% of children with HIV have poor linear growth.^1^ In sub-Saharan Africa, where 90% of the world's children with HIV live, high background rates of malnutrition and intercurrent infections further impair linear growth, such that the prevalence and extent of stunting is markedly higher than in high-income settings.^1^, ^2^ Puberty is a crucial period for bone mass accrual; after cessation of linear growth, consolidation of mineral continues until peak bone mass (PBM) is reached in early adulthood, serving as a reservoir of bone for later life. Therefore, disturbances in growth or pubertal delay due to HIV infection have implications for bone mass accrual and PBM. Low PBM is a principal determinant of subsequent adult osteoporotic fracture risk; a 10% reduction in PBM doubles fracture risk in adulthood.^3^

The scale-up of antiretroviral therapy (ART) programmes globally has substantially improved the survival of people with HIV, and increasing numbers of children with HIV—who would otherwise have died in early childhood—are surviving to adolescence and adulthood.^4^ Catch-up linear growth occurs once ART is initiated, but individuals who start ART in late childhood might not realise their full growth potential or attain population age growth norms.^5^ In sub-Saharan Africa, children start ART later than in high-income settings, at 7·9 years (IQR 6·0–9·3) compared with 0·9 years (IQR 0·4–2·6) in North America.^6^ Furthermore, the prevalence of stunting is higher in sub-Saharan Africa, meaning HIV might have a more detrimental effect on skeletal growth than in high-income settings.^2^ Notably, small cross-sectional studies from South Africa have shown reduced bone mass^7^ and strength^8^ in mostly prepubertal children with HIV.

**Research in context**

**Evidence before this study**

We and others have previously shown that faltered linear growth resulting in stunting is common in children with HIV in sub-Saharan Africa. Successful roll-out of antiretroviral therapy (ART) programmes has enabled increased numbers of children to survive adolescence and reach adulthood. Puberty is a crucial period of skeletal development, during which bone mass accrues to achieve, by early adulthood, peak bone mass, which has been shown to be a key determinant of lifetime fracture risk. We searched Embase, Ovid MEDLINE(R) (including Epub Ahead of Print and Daily databases), Cochrane Library, and conference proceedings (through Web of Science) from inception until March 10, 2021, adapting search strategies for each database to include combinations of medical subject headings (MeSH terms) relevant to bone growth, development, HIV, and ART in children, without language restrictions. Few studies had investigated the effect of HIV on adolescent skeletal growth in low-income settings and even fewer had adequately considered the effect of skeletal size on bone density measurement techniques; consideration of skeletal size is crucial as the most commonly used measure, dual energy X-ray absorptiometry (DXA), underestimates bone density when skeletons are small (as is the case with stunting). The International Society for Clinical Densitometry (ISCD) has recommended specific size-adjustment techniques to overcome this problem. These techniques had not been used to understand the effect of HIV on bone mass accrual in populations in sub-Saharan Africa.

**Added value of this study**

To our knowledge, this study is the largest to investigate the effect of HIV infection on skeletal health in children (aged 8–16 years) in sub-Saharan Africa. Our findings are strengthened by use of the ISCD-recommended size-adjustment methods for DXA data, which was particularly important given the observed height differences (levels of stunting) in our study population. We found that marked deficits in bone density were common in children with HIV, who had a substantially higher prevalence of low bone density (a deficit of 2 SDs or more) than their HIV-uninfected peers. The effect of HIV on bone density was most marked in the late stages of puberty, especially in girls. Use of tenofovir disoproxil fumarate (TDF) was strongly associated with bone deficits, particularly affecting the total body (predominantly reflecting cortical bone). TDF exposure for 4 or more years was associated with a 0·52 SD deficit in total body Z-score, which translates clinically to an approximately 50% increase in fracture risk in childhood and, if sustained, adulthood.

**Implications of all the available evidence**

This study highlights the importance of addressing the long-term adverse effects of HIV infection on musculoskeletal health in children living in sub-Saharan Africa. If the type of bone deficits we identified persist into adulthood, this African region is at risk of increased fracture incidence, particularly within the current birth cohort. The finding that bone mineral deficits appear greatest at the end of puberty in girls is of concern, as girls are likely to depend on potentially inadequate skeletal calcium reserves to support pregnancy and lactation in adulthood, which might increase their risk of vertebral fracture. TDF is currently one of the recommended component drugs of a first-line ART regimen in children of sufficient age and weight (as well as in adults), and is thus extensively used in sub-Saharan Africa, sometimes even in children who do not quality for its use when other options are not available. The detrimental effects associated with TDF use in this study support re-evaluation of its first-line use during skeletal growth.

Most studies investigating the effect of HIV infection on skeletal growth have been done in high-income settings and have not taken into account the effect of poor growth on bone density.^9^ Dual energy X-ray absorptiometry (DXA) is commonly used to measure bone density; however, when skeletons are small (in the case of HIV, due to stunting), DXA underestimates bone density,^10^ and therefore adjustment for skeletal size is important in paediatric populations. Our aim was to understand the relationship between HIV infection and skeletal health in peripubertal children on ART in Zimbabwe, as a country with a generalised and sustained severe HIV epidemic. Specifically, we sought to identify the prevalence of low bone density adjusted for skeletal size in children with and without HIV, and to investigate risk factors associated with reduced size-adjusted bone density.

## Methods

### Study design and participants

We did a cross-sectional study using baseline DXA bone measurements from the IMVASK study, which is a prospective cohort study on the impact of vertical HIV infection on child and adolescent skeletal development in Harare, Zimbabwe. The IMVASK protocol has been published elsewhere (ISRCTN registry ISRCTN12266984)^11^ and 12-month follow-up has been completed. Children aged 8–16 years with HIV were recruited from outpatient HIV clinics at the only two public sector general hospitals in Harare (Parirenyatwa General Hospital and Harare Central Hospital). Studies in children suggest that ART initiation is followed by an initial decrease in bone mass, which stabilises after 2 years,^12^ and therefore we enrolled children with HIV who had been taking ART for at least 2 years. Systematic quota-based sampling, stratified by age and sex, was used to recruit 50 male children and 50 female children in each of three age groups (8–10 years, 11–13 years, and 14–16 years). Exclusion criteria were being acutely unwell (defined as requiring immediate hospitalisation), not residing in Harare, and being unaware of one's HIV status (to avoid inadvertent disclosure during study participation). A maximum of five children with HIV were recruited each day for logistical reasons.

A comparison group of children without HIV was recruited from six government primary and secondary schools randomly selected from the 109 primary schools and 44 secondary schools within the same suburbs in Harare where the hospitals provide HIV care. A random number sequence was computer-generated and applied to a list provided by the Ministry of Primary and Secondary Education of all schools in the area. Schools were approached in sequence to seek consent. Schools which declined to participate were replaced by schools on a reserve list until the target of six schools was reached. Younger children (8–12 years) were sampled from primary schools and older children (14–16 years) from secondary schools, with children aged 13 years sampled from both schools. The number of children selected from each school was proportional to school size, thereby giving each child equal probability of being sampled. We applied a random number sequence to school registers to select participants using the same quota-based sampling approach of 50 male children and 50 female children in each of the three age strata. Letters were sent to the households of children who had been randomly selected. Children underwent HIV testing after enrolment; those testing positive and not in care were referred to HIV services. Children in schools who tested positive were considered for enrolment in the HIV cohort.

Ethical and governance approvals were granted by the ethics committee of the London School of Hygiene & Tropical Medicine (London, UK; reference 15333), the institutional review board of the Biomedical Research and Training Institute in Harare (reference AP145/2018), the joint research ethics committee for the University of Zimbabwe College of Health Sciences (Harare) and the Parirenyatwa Group of Hospitals (Harare; reference 11/18), the Harare Central Hospital ethics committee (reference 170118/04), the Medical Research Council of Zimbabwe (Harare; reference MRCZ/A/2297), and the Ministry of Primary and Secondary Education of the Government of Zimbabwe (Harare; reference C/426/Harare). Parents or guardians provided written informed consent for study participation and HIV testing, and children provided written assent.

### Procedures

All data in this study were collected at recruitment (baseline) in the IMVASK study, from May 4, 2018, to Jan 21, 2020. A questionnaire administered by research staff was used to collect sociodemographic and clinical data, including smoking status and alcohol and steroid use, from children in the company of a parent or guardian (with parents and guardians allowed to answer questions on the child's behalf). The International Physical Activity Questionnaire, validated in multiple countries including South Africa but not Zimbabwe, was used to assess physical activity as multiples of the resting metabolic rate (MET) in MET minutes. Diet and nutrition were assessed with a tool that was based on a validated dietary diversity and food frequency tool from India and Malawi,^13^ and adapted to the Zimbabwean context with international guidelines applicable to sub-Saharan Africa.^14^ This tool quantified dietary calcium and vitamin D intake plus sunlight exposure; adaptations reflected the Zimbabwean context in which fortification of oils and margarine with vitamin D is mandated and specific vitamin D rich foods, such as Kapenta fish, are commonly eaten.

Anthropometric measurements were done by trained research nurses and research assistants at the study clinics. Standing and sitting height, which was measured to the nearest 0·1 cm (with a Seca 213 stadiometer; Seca, Hamburg, Germany), and weight, which was measured to the nearest 0·1 kg (with Seca 875 weight scales), were recorded by two separate readers. If height measurements differed by more than 0·5 cm, or weight measurements by more than 0·5 kg, a third reading was taken by an additional reader, and final height and weight values were taken as means of the two or three measurements. The same staff measured the children with and without HIV. All equipment was calibrated annually. Tanner pubertal staging was done by a nurse and doctor, with an orchidometer used to assess testicular volume in males. Pubertal delay was defined as not having reached Tanner stage 2 in girls aged 13 years or older, and in boys aged 14 years or older.

Details collected for participants with HIV were age at HIV diagnosis, probable mode of transmission, ART regimen and duration, and current CD4 cell count and HIV viral load. CD4 cell count was measured with an Alere PIMA CD4 Analyser (Waltham, MA, USA) and HIV viral load with the GeneXpert HIV-1 Viral Load assay (Cepheid, Sunnyvale, CA, USA), with viral suppression defined as fewer than 1000 HIV RNA copies per ml (as per WHO guidelines).

DXA scans of the lumbar spine and total body were done by one of two trained radiographers according to standard procedures on a Hologic QDR Wi densitometer (Hologic, Bedford, MA, USA) with Apex software (version 4.5)^15^ for scan analysis. Daily calibration was done with the manufacturer-provided spine phantom. DXA scans were repeated in a subgroup (n=30) selected by convenience sampling to confirm reproducibility. The precision error was a root mean square deviation of 0·011 g/cm^2^ (lumbar spine) and 0·010 g/cm^2^ (total body) with a coefficient of variation of 1·35% (lumbar spine) and 1·22% (total body). As mentioned, an important limitation of DXA in paediatric populations with chronic disease is that the two-dimensional (areal) bone density values are highly dependent on bone size; thus DXA underestimates bone density in small children.^10^ We therefore used the two main size-adjustment techniques recommended by the International Society for Clinical Densitometry (ISCD) to overcome the size dependence of DXA measurement:^16^ we measured total-body less-head bone mineral content for lean mass adjusted for height (TBLH-BMC^LBM^), and lumbar spine bone mineral apparent density (LS-BMAD). LS-BMAD was calculated from DXA-measured lumbar spine data with the Carter method.^17^ TBLH-BMC^LBM^ was calculated from the whole body scan with published derived equations for Hologic DXA scans, which adjust for log-transformed total body lean mass, total body fat mass, height, and age.^18^ Sex-matched and age-matched Z-scores were generated with Hologic UK population reference data from 1996–2012 in 4–20-year-olds as recommended by ISCD guidelines, as no local reference data were available.^16^ Low TBLH-BMC^LBM^ and LS-BMAD were defined by a Z-score of less than −2·0.^18^

### Statistical analysis

A sample size of 300 in each group was required to permit detection of a difference between children with and without HIV in DXA-measured size-adjusted bone density Z-scores of 0·23, with 80% power and a significance level of 0·05 assuming an SD of 1·3.^19^ The study had 80% power to detect a 4·8% difference in prevalence of low TBLH-BMC^LBM^ between children with and without HIV, assuming a prevalence in those without HIV of 1·0%.^20^

Height-for-age and weight-for-age Z-scores were calculated with 1990 UK reference data,^21^ with Z-scores of less than −2·0 defining stunting (height-for-age score) and underweight (weight-for-age score). We derived socioeconomic status using the first component from a principal component analysis combining an asset list (detailing number in the household, head of household age, highest maternal and paternal education levels, household ownership, monthly household income, access to amenities [electricity, water, and a flush toilet or pit latrine], and household item ownership [fridge, bicycle, car, and television or radio]). Socioeconomic status was split into tertiles (low, middle, and high) for analysis.

Analyses were done with Stata (version 16.1). The primary exposure was HIV, and primary outcomes were TBLH-BMC^LBM^ and LS-BMAD Z-scores. We compared participant characteristics between those with and without HIV using independent sample t-tests for means, Wilcoxon signed-rank tests for non-parametric variables, and χ^2^ or Fisher's exact tests for proportions. The same methods were used to compare children with and without missing data. To first understand the role of sex, puberty, and HIV on bone outcomes, we examined mean difference in TBLH-BMC^LBM^ and LS-BMAD Z-scores between participants with and without HIV using linear regression with robust standard errors, overall and stratified by sex, Tanner stage (stages 1 and 2 *vs* stages 3–5), and age group (8–10 years, 11–13 years, and 14–16 years). Similarly, we examined mean risk difference for low TBLH-BMC^LBM^ and low LS-BMAD Z-scores (less than −2 *vs* −2 or higher) using generalised linear models, with Poisson distributions and log links with robust standard errors. The models were adjusted for age, sex, and pubertal stage. Mean differences were estimated with 95% CIs. Three-way interactions between sex, pubertal stage, and HIV were assessed with Wald tests in linear regression models. In secondary analyses of absolute measures of TBLH-BMC^LBM^ and LS-BMAD, generalised linear models with log link and gamma distribution were used, and marginal means and marginal mean differences with 95% CIs estimated.

Associations between potential risk factors and TBLH-BMC^LBM^ and LS-BMAD Z-scores were investigated with linear regression separately for participants with and without HIV. Adjustment was made for a priori confounders (age, sex, and pubertal stage^22^), potential risk factors (socioeconomic status,^23^ physical activity,^24^ calcium and vitamin D intake,^25^ and, in those with HIV, CD4 cell count, viral load, tenofovir disoproxil fumarate [TDF] exposure, and age at ART initiation^26^), variables associated in complete case analysis with TBLH-BMC^LBM^ or LS-BMAD Z-score (at p<0·20), and variables associated with missing status.

All enrolled children were included in the analyses. To account for missing data, including DXA-measured outcomes, we used multiple imputation by chained equations with seven imputed datasets, which allowed for imputation of categorical and continuous data jointly. It was assumed that data were missing at random. Our imputation models included all outcomes, auxiliary variables associated with missingness and with study group (with or without HIV), and variables identified in complete case analysis to be associated with TBLH-BMC^LBM^ or LS-BMAD Z-score (p<0·20). In all analyses, the significance level was 0·05.

### Role of the funding source

The funder of the study had no role in study design, data collection, data analysis, data interpretation, or writing of the report, or in the decision to submit the paper for publication.

## Results

**Figure 1:**
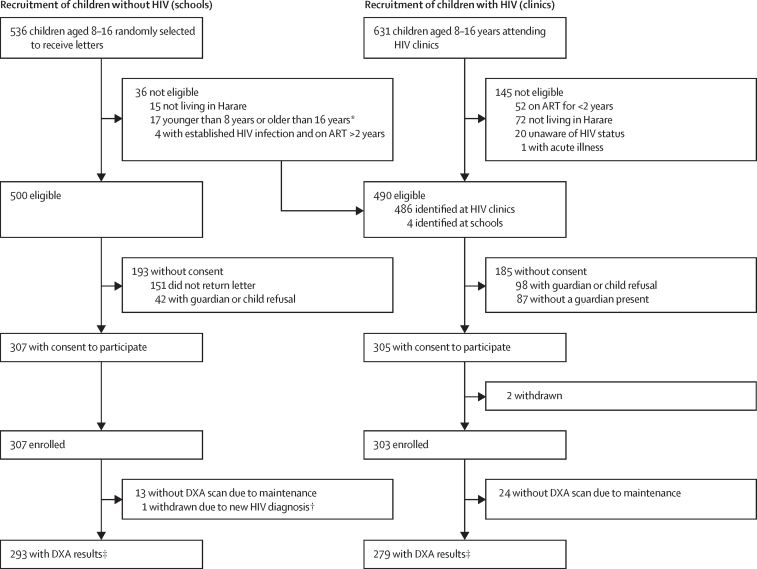
Recruitment of study participants ART=antiretroviral therapy. DXA=dual-energy X-ray absorptiometry. *Handwritten class registers were used to identify children and sometimes listed an incorrect date of birth; therefore some children outside the 8–16 years age range were sent letters. †Children underwent HIV testing after enrolment. ‡All enrolled children were included in the final analyses unless withdrawn from the study; when children had missing data, multiple imputation methods were used.

Among 631 children with HIV attending clinics, 486 (77%) were eligible for enrolment. Four additional children with HIV, identified from screening in schools, met the inclusion criteria. Of these 490 eligible children, 303 (62%) were enrolled (figure 1). For most children with HIV who were eligible but did not enrol, the reason was refusal by the child or guardian, or no guardian being present. Compared with these children with HIV who were eligible but not enrolled, study participants with HIV were a mean of 1·2 years (SE 0·3) older, more commonly girls (151 [50%] of 303 enrolled *vs* 75 [40%] of 187 excluded), and more likely to attend school (295 [97%] *vs* 168 [90%]). Of 536 children randomly selected from schools, 500 (93%) were eligible, of whom 307 (61%) consented to participate. One was excluded after testing HIV-positive, giving a total of 306 participants without HIV (mean age 12·5 [2·5] years; 155 [51%] girls). Compared with children without HIV who were eligible but not enrolled, study participants without HIV were a mean of 0·6 years (SE 0·2) older. Sex distribution was similar (by number of girls, 155 [51%] of 306 enrolled *vs* 95 [49%] of 194 excluded). The mean age of enrolled children with HIV was 12·4 years [SD 2·5], and 151 [50%] were girls. The mean age of enrolled children without HIV was 12·5 years [2·5], and 155 [51%] were girls (table 1).

**Table 1:** Characteristics of study participants

		**HIV-positive (n=303)**	**HIV-negative (n=306)**	**p value**
**Sociodemographic factors**				
Age, years		303; 12·4 (2·5)	306; 12·5 (2·5)	0·69
Female sex		303; 151 (50%)	306; 155 (51%)	0·84
Socioeconomic status				
	Low: tertile 1	303; 115 (38%)	306; 88 (29%)	..
	Middle: tertile 2	303; 105 (35%)	306; 98 (32%)	..
	High: tertile 3	303; 83 (27%)	306; 120 (39%)	0·0051
Orphanhood (one or both parents dead)		290; 123 (42%)	303; 20 (7%)	<0·0001
Past or current tuberculosis		302; 49 (16%)	305; 2 (1%)	<0·0001
**Lifestyle factors**				
Outdoor time, >2 h/day		303; 224 (74%)	306; 171 (56%)	<0·0001
Physical activity level				
	Low: <600 MET minutes per week	303; 148 (49%)	306; 114 (37%)	..
	Moderate: 600–3000 MET minutes per week	303; 77 (25%)	306; 88 (29%)	..
	High: >3000 MET minutes per week	303; 78 (26%)	306; 104 (34%)	0·012
Daily dietary calcium intake				
	Very low: <150 mg/day	303; 135 (45%)	306; 136 (44%)	..
	Low: 150–299 mg/day	303; 62 (20%)	306; 66 (22%)	..
	Moderate: 300–450 mg/day	303; 106 (35%)	306; 104 (34%)	0·94
Daily dietary vitamin D intake				
	Very low: <4·0 μg/day	303; 40 (13%)	306; 37 (12%)	..
	Low: 4·0–5·9 μg/day	303; 205 (68%)	306; 197 (64%)	..
	Moderate: 6·0–7·9 μg/day	303; 58 (19%)	306; 72 (24%)	0·41
**Puberty characteristics**				
Pubertal stage				
	Tanner 1	287; 117 (41%)	303; 69 (23%)	..
	Tanner 2	287; 59 (21%)	303; 69 (23%)	..
	Tanner 3	287; 55 (19%)	303; 53 (17%)	..
	Tanner 4	287; 44 (15%)	303; 92 (30%)	..
	Tanner 5	287; 12 (4%)	303; 20 (7%)	<0·0001
Pubertal delay*		127; 8 (6%)	132; 0	0·0034
**HIV characteristics**				
Age at HIV diagnosis, years		303; 3·0 (1·2–5·8)	NA	..
Age at ART initiation, years		303; 3·7 (1·8–6·9)	NA	..
ART duration, years		303; 8·1 (6·2–9·5)	NA	..
Proportion of life on ART, %		303; 65·4% (22·1)	NA	..
Current tenofovir disoproxil fumarate use		303; 102 (34%)	NA	..
Viral load <1000 RNA copies per ml		268; 212 (79%)	NA	..
CD4 count <500 cells per μL		288; 58 (20%)	NA	..
**Anthropometry**				
Standing height Z-score		302; −1·68 (1·23)	306; −0·63 (1·08)	<0·0001
Height-for-age Z-score less than −2†		302; 97 (32%)	306; 24 (8%)	<0·0001
Sitting height for age Z-score		303; −2·11 (1·12)	303; −1·38 (1·21)	<0·0001
Sitting height-for-age Z-score less than −2		303; 152 (50%)	303; 76 (25%)	<0·0001
Weight-for-age Z-score		303; −1·46 (1·20)	304; −0·55 (1·24)	<0·0001
Weight-for-age Z-score less than −2‡		303; 79 (26%)	304; 26 (9%)	<0·0001
BMI Z-score		302; −0·63 (1·01)	304; −0·28 (1·17)	<0·0001
BMI Z-score less than −2		302; 28 (9%)	304; 18 (6%)	0·12
**Bone density measures**				
TBLH-BMC^LBM^, g		279; 950·9 (272·8)	292§; 1090·3 (336·1)	<0·0001
TBLH-BMC^LBM^ Z-score		279; −0·61 (1·08)	292§; −0·41 (1·00)	0·018
TBLH-BMC^LBM^ Z-score less than −2		279; 29 (10%)	292§; 18 (6%)	0·066
LS-BMAD, g/cm^3^		279; 0·202 (0·035)	293; 0·210 (0·037)	0·013
LS-BMAD Z-score		279; −0·51 (1·40)	293; −0·24 (1·21)	0·014
LS-BMAD Z-score less than −2		279; 40 (14%)	293; 17 (6%)	0·0007

Data are available sample; n (%), available sample; mean (SD), or available sample; median (IQR). MET=resting metabolic rate. ART=antiretroviral therapy. BMI=body-mass index. TBLH-BMC^LBM^=total body less head bone mineral content for lean mass adjusted for height. LS-BMAD=lumbar spine bone mineral apparent density.

* Definition of pubertal delay (lower than Tanner stage 2) applicable for girls aged 13 years or older and boys aged 14 years or older.

† Defined as stunted growth.

‡ Defined as underweight.

§ Missing data from one participant who had both lumbar spine and total body scans but the total body scan could not be analysed due to a software analysis error.

Compared with children without HIV, a significantly higher proportion of children with HIV were of low socioeconomic status, were orphaned, had past or current tuberculosis, were less physically active, and reported spending more time outdoors (table 1). Across the whole study population, consumption of vitamin D and calcium was low, with 479 (79%) of 609 children consuming less than 6·0 μg/day vitamin D (US National Institutes of Health recommended daily allowance [RDA] ≥15 μg/day), and 399 (66%) consuming less than 300 mg/day of calcium (RDA ≥1300 mg/day). No participants reported smoking, drinking alcohol, or oral steroid use at the time of enrolment. The 24 children with HIV and 14 children without HIV who were missing DXA data (figure 1) were thinner (based on BMI Z-score <2) and shorter (based on standing height Z-score) than those with DXA data (appendix p 1). Additionally, orphanhood status and earlier pubertal stage were associated with missingness of data when considering all outcomes and covariates (appendix pp 1–2).

Five (2%) of the 303 children with HIV were not perinatally infected. The median age of HIV diagnosis was 3·0 years (IQR 1·2–5·8), with ART initiated at a median age of 3·7 years (1·8–6·9; table 1). Median ART duration was 8·1 (IQR 6·2–9·5) years. At enrolment, 102 (34%) of 303 children were on a TDF regimen, with a median duration of TDF use of 3·0 years (IQR 1·4–5·5). Overall, 211 children (70%) were taking a non-nucleoside reverse-transcriptase inhibitor ART regimen, and 89 (29%) a protease inhibitor ART regimen. The median CD4 count was 766 cells per μl (IQR 537–1019), and 212 children (79%) had a suppressed HIV viral load (table 1).

Compared with children without HIV, a significantly higher proportion of children with HIV had stunted growth (97 [32%] of 302 with available data vs 24 [8%] of 306; p<0·0001) and were underweight (79 [26%] of 303 *vs* 26 [9%] of 304 with available data; p<0·0001; table 1). The height differences between children with and without HIV were greater for standing height than for sitting height (mean Z-score difference 1·05 [95% CI 0·87–1·24] for standing height and 0·73 [0·55–0·92] for sitting height). Compared with children without HIV, children with HIV were more likely to be at an earlier Tanner stage, and, among older girls (≥13 years) and older boys (≥14 years), to have pubertal delay (8 [6%] of 127 with available data *vs* 0 of 132 with available data; p=0·0034; table 1; appendix p 3).

DXA-based measures were available in 279 children with HIV and 293 children without HIV (figure 1). In children with HIV, LS-BMAD was available in 293 children and TBLH-BMC^LBM^ in 292 (table 1). Mean TBLH-BMC^LBM^ and LS-BMAD, in terms of absolute values and Z-scores, were lower in children with HIV than in those without HIV. Children with HIV had a higher prevalence of low TBLH-BMC^LBM^ Z-score than children without HIV (29 [10%] of 279 *vs* 18 [6%] of 292; p=0·066) and a higher prevalence of low LS-BMAD Z-score (40 [14%] of 279 *vs* 17 [6%] of 293; p=0·0007; table 1). Overall, children with HIV had a 0·20 (95% CI 0·03–0·37) lower TBLH-BMC^LBM^ Z-score and 0·26 (0·04–0·48) lower LS-BMAD Z-score than children without HIV, in terms of crude mean difference (table 2). Differences in LS-BMAD Z-score were similar in girls and boys, whereas differences in TBLH-BMC^LBM^ Z-score were more apparent in girls. Both boys and girls with HIV had an increased risk of low LS-BMAD Z-score, with boys in early puberty showing the greatest risk of low Z-score (table 2). In children with HIV, low LS-BMAD was associated with stunting; this association was not observed in children without HIV (appendix p 4).

**Table 2:** Mean difference in Z-scores and risk difference in low Z-scores between children with and without HIV

			n*	**Crude mean difference**†**(Z-scores) or crude risk difference**‡**(low Z-scores)**	**Adjusted mean difference**†§**(Z-scores) or adjusted risk difference**‡§**(low Z-scores)**
**TBLH-BMC**^LBM^**Z-score**					
Overall¶			609	−0·20 (−0·37 to −0·03)	−0·13 (−0·31 to 0·05)
Boys‖			303	−0·12 (−0·36 to 0·11)	−0·06 (−0·30 to 0·18)
Girls‖			306	−0·27 (−0·52 to −0·02)	−0·20 (−0·46 to 0·06)
Boys					
	Tanner stage 1–2**		175	−0·18 (−0·49 to 0·12)	−0·17 (−0·47 to 0·14)
	Tanner stage 3–5**		117	−0·12 (−0·50 to 0·26)	−0·11 (−0·50 to 0·27)
Girls					
	Tanner stage 1–2**		139	−0·14 (−0·50 to 0·22)	−0·12 (−0·48 to 0·23)
	Tanner stage 3–5**		159	−0·35 (−0·71 to 0·01)	−0·34 (−0·70 to 0·03)
**LS-BMAD Z-score**					
Overall¶			609	−0·26 (−0·48 to −0·04)	−0·11 (−0·32 to 0·11)
Boys‖			303	−0·27 (−0·58 to 0·04)	−0·17 (−0·46 to 0·13)
Girls‖			306	−0·23 (−0·51 to 0·04)	−0·04 (−0·32 to 0·24)
Boys					
	Tanner stage 1–2**		175	−0·29 (−0·68 to 0·10)	−0·16 (−0·54 to 0·22)
	Tanner stage 3–5**		117	−0·44 (−0·95 to 0·07)	−0·46 (−0·95 to 0·03)
Girls					
	Tanner stage 1–2**		139	−0·09 (−0·53 to 0·34)	0·00 (−0·42 to 0·42)
	Tanner stage 3–5**		159	−0·30 (−0·66 to 0·07)	−0·24 (−0·61 to 0·13)
**TBLH-BMC**^LBM^**low Z-score (less than −2)**					
Overall¶			609	0·04 (−0·005 to 0·08)	0·01 (−0·03 to 0·05)
Boys‖			303	0·05 (−0·01 to 0·11)	0·02 (−0·03 to 0·07)
Girls‖			306	0·02 (−0·04 to 0·08)	0·00 (−0·04 to 0·05)
	Boys				
		Tanner stage 1–2**	175	0·04 (−0·03 to 0·12)	0·04 (−0·05 to 0·13)
		Tanner stage 3–5**	117	0·05 (−0·05 to 0·16)	0·04 (−0·04 to 0·12)
	Girls				
		Tanner stage 1–2**	139	0·00 (−0·09 to 0·10)	−0·01 (−0·13 to 0·12)
		Tanner stage 3–5**	159	0·03 (−0·04 to 0·11)	0·02 (−0·04 to 0·09)
**LS-BMAD low Z-score (less than −2)**					
Overall¶			609	0·08 (0·03 to 0·12)	0·02 (−0·02 to 0·06)
Boys‖			303	0·09 (0·01 to 0·17)	0·03 (−0·03 to 0·09)
Girls‖			306	0·06 (0·01 to 0·11)	0·02 (−0·01 to 0·05)
	Boys				
		Tanner stage 1–2**	175	0·13 (0·05 to 0·22)	0·14 (0·04 to 0·24)
		Tanner stage 3–5**	117	0·07 (−0·09 to 0·23)	0·04 (−0·06 to 0·13)
	Girls				
		Tanner stage 1–2**	139	0·08 (0·00 to 0·17)	0·09 (−0·03 to 0·21)
		Tanner stage 3–5**	159	0·03 (−0·03 to 0·08)	0·01 (−0·02 to 0·04)

Values in parentheses are 95% CIs. Negative mean difference values indicate a lower mean in children with HIV; positive risk difference values indicate an increased risk of low Z-score (less than −2·0) in children with HIV. TBLH-BMC^LBM^=total body less head bone mineral content for lean mass adjusted for height. LS-BMAD=lumbar spine bone mineral apparent density.

* Non-missing data; the missing data for TBLH-BMC^LBM^ Z-score (19 boys and 19 girls), LS-BMAD Z-score (18 boys and 19 girls), pubertal stage (11 boys and eight girls), and orphanhood (nine boys and seven girls) were estimated with multiple imputation models and numbers in each pubertal stage stratum varied by imputation dataset.

† Linear regression with robust standard errors.

‡ Generalised linear model, Poisson distribution, and log link with robust standard errors.

§ Model adjusted for age, sex, and pubertal stage.

¶ Tanner stage included as a variable with five levels.

‖ Estimated by fitting an interaction term for sex by HIV status; in models adjusted for Tanner stage (as five levels).

** Estimated by fitting a three-way interaction term for sex by Tanner stage (two categories) and HIV status.

**Figure 2:**
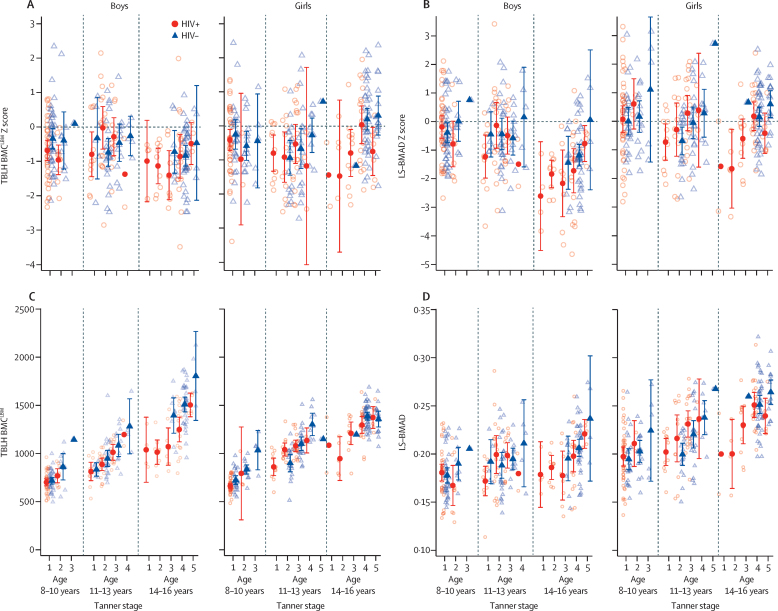
Mean values for (A) TBLH-BMC^LBM^ Z-score, (B) LS-BMAD Z-score, (C) absolute TBLH-BMC^LBM^, and (D) absolute LS-BMAD, stratified by sex, age, pubertal stage, and HIV status Unadjusted data are shown. Error bars indicate 95% CIs. Raw data are presented as open circles (HIV-positive) and open triangles (HIV-negative). TBLH-BMC^LBM^=total body less head bone mineral content for lean mass adjusted for height. LS-BMAD=lumbar spine bone mineral apparent density.

In each age group (8–10 years, 11–13 years, and 14–16 years), both male and female children with HIV were at an earlier Tanner stage than their HIV-negative peers of the same sex (appendix p 3). Mean absolute values of TBLH-BMC^LBM^ and LS-BMAD differed by pubertal stage and sex (three-way interactions: TBLH-BMC^LBM^ p=0·041, LS-BMAD p=0·88), such that differences in absolute and Z-score values for TBLH-BMC^LBM^ and LS-BMAD between participants with and without HIV were most marked in the late pubertal stages (figure 2). Differences in size-adjusted bone density between participants with and without HIV were generally small in the early stages of puberty (table 2). In the later stages of puberty, differences in bone outcomes between those with and without HIV were more apparent for both sexes, except for TBLH-BMC^LBM^ Z-score in boys. Girls particularly showed evidence of differences in TBLH-BMC^LBM^ and LS-BMAD for Z-scores and absolute values (figure 2, table 2). Stratification by age group, sex, and pubertal stage caused wide confidence intervals (figure 2) as few older participants with HIV (14–16 years) were in the late stages of puberty (Tanner stages 4 and 5), compared with participants of the same age without HIV (appendix p 3). When models assessing differences in TBLH-BMC^LBM^ and LS-BMAD Z-scores between children with and without HIV were adjusted for age, sex, and pubertal stage, the Z-score differences were marginally decreased (−0·13 [95% CI −0·31 to 0·05] for TBLH-BMC^LBM^ Z-score and −0·11 [–0·32 to 0·11] for LS-BMAD Z-score). However, even after adjustment for age, male children with HIV in the early stages of puberty had a greater risk of low LS-BMAD than did males without HIV (risk difference 0·14 [95% CI 0·04–0·24]; table 2).

Among children without HIV, male sex (*vs* female sex) and earlier pubertal stage (*vs* later pubertal stage) were associated with lower TBLH-BMC^LBM^ and LS-BMAD Z-scores in crude analysis, but not in the adjusted analysis (appendix pp 5–6). Among children with HIV, being an orphan (vs not an orphan) and TDF exposure (*vs* non-exposure) were associated with lower TBLH-BMC^LBM^ Z-score in unadjusted analysis and after adjustment for age, sex, pubertal stage, socioeconomic status, physical activity, calcium and vitamin D intake, CD4 cell count, HIV viral load, age at ART initiation, orphanhood status, and TDF exposure (table 3). After adjustment, TDF exposure for 4 or more years (*vs* no exposure) was associated with a 0·52 (95% CI 1·04 to 0·003; p=0·046) SD deficit in TBLH-BMC^LBM^ Z-score, and orphanhood with a 0·46 (0·75 to 0·17; p=0·0023) SD deficit.

**Table 3:** Characteristics associated with TBLH-BMC^LBM^ Z-score in participants with HIV

	n (total N=279)*	**Mean TBLH-BMC^LBM^ Z-score**†	**Crude β coefficient (95% CI)**‡	**p value**	**Adjusted β coefficient (95% CI)**‡§	**p value**
**Age**						
8–10 years	94	−0·58	1 (ref)	..	1 (ref)	..
11–13 years	94	−0·48	0·13 (−0·18 to 0·43)	..	0·16 (−0·27 to 0·58)	..
14–16 years	91	−0·78	−0·16 (−0·48 to 0·15)	0·20	−0·15 (−0·73 to 0·44)	0·32
**Sex**						
Male	142	−0·69	1 (ref)	..	1 (ref)	..
Female	137	−0·53	0·14 (−0·13 to 0·40)	0·30	0·12 (−0·14 to 0·38)	0·36
**Pubertal stage**						
Tanner 1	107	−0·63	1 (ref)	..	1 (ref)	..
Tanner 2	56	−0·74	−0·07 (−0·43 to 0·29)	..	0·08 (−0·32 to 0·49)	..
Tanner 3	49	−0·67	−0·02 (−0·37 to 0·33)	..	0·02 (−0·46 to 0·51)	..
Tanner 4	40	−0·51	0·12 (−0·34 to 0·58)	..	0·45 (−0·18 to 1·08)	..
Tanner 5	12	−0·64	−0·05 (−0·70 to 0·59)	0·95	0·20 (−0·60 to 1·01)	0·52
**Socioeconomic status**						
High: tertile 3	78	−0·62	1 (ref)	..	1 (ref)	..
Middle: tertile 2	93	−0·64	−0·02 (−0·35 to 0·30)	..	0·03 (−0·26 to 0·39)	..
Low: tertile 1	108	−0·58	0·05 (−0·27 to 0·38)	0·87	0·06 (−0·26 to 0·39)	0·93
**Orphanhood**						
Not an orphan	154	−0·42	1 (ref)	..	1 (ref)	..
One or both parents dead	113	−0·84	−0·45 (−0·71 to −0·18)	0·0011	−0·46 (−0·75 to −0·17)	0·0023
**Physical activity level**						
High: >3000 MET minutes per week	76	−0·53	1 (ref)	..	1 (ref)	..
Moderate: 600–3000 MET minutes per week	69	−0·62	−0·09 (−0·43 to 0·26)	..	0·02 (−0·34 to 0·37)	..
Low: <600 MET minutes per week	134	−0·65	−0·13 (−0·44 to 0·18)	0·71	−0·03 (−0·35 to 0·29)	0·96
**Daily dietary calcium intake**						
Moderate: 300–450 mg/day	95	−0·70	1 (ref)	..	1 (ref)	..
Low: 150–299 mg/day	60	−0·64	0·07 (−0·28 to 0·42)	..	0·11 (−0·26 to 0·47)	..
Very low: <150 mg/day	124	−0·53	0·20 (−0·09 to 0·48)	0·39	0·24 (−0·06 to 0·53)	0·30
**Daily dietary vitamin D intake**						
Moderate: 6·0–7·9 μg/day	50	−0·40	1 (ref)	..	1 (ref)	..
Low: 4·0–5·9 μg/day	190	−0·62	−0·18 (−0·51 to 0·15)	..	−0·25 (−0·58 to 0·09)	..
Very low: <4·0 μg/day	39	−0·82	−0·38 (−0·83 to 0·06)	0·25	−0·50 (−0·96 to −0·03)	0·11
**CD4 count**						
≥500 cells per μL	213	−0·59	1 (ref)	..	1 (ref)	..
<500 cells per μL	54	−0·70	−0·08 (−0·40 to 0·24)	0·64	−0·07 (−0·42 to 0·28)	0·71
**HIV viral load**						
≥1000 RNA copies per ml	54	−0·53	1 (ref)	..	1 (ref)	..
<1000 RNA copies per ml	207	−0·65	−0·16 (−0·47 to 0·15)	0·30	−0·24 (−0·60 to 0·12)	0·19
**Age at ART initiation**						
<4 years	148	−0·61	1 (ref)	..	1 (ref)	..
4–8 years	83	−0·58	0·04 (−0·27 to 0·35)	..	0·11 (−0·23 to 0·45)	..
>8 years	48	−0·68	−0·02 (−0·37 to 0·33)	0·94	0·21 (−0·21 to 0·63)	0·62
**TDF years of exposure**						
None	184	−0·48	1 (ref)	..	1 (ref)	..
<4 years	59	−0·80	−0·30 (−0·61 to 0·02)	..	−0·35 (−0·69 to 0·0003)	..
≥4 years	36	−0·95	−0·48 (−0·93 to −0·02)	0·037	−0·52 (−1·04 to −0·003)	0·046

TBLH-BMC^LBM^=total body less head bone mineral content for lean mass adjusted for height. MET=resting metabolic rate. ART=antiretroviral therapy. TDF=tenofovir disoproxil fumarate.

* Non-missing data.

† Arithmetic mean of non-missing data.

‡ β coefficient from linear regression indicates the Z-score difference from the reference category; lower values indicate lower Z-scores; missing data for TBLH-BMC^LBM^ Z-score (n=24), lumbar spine bone mineral apparent density Z-score (n=24), pubertal stage (n=16), orphanhood (n=13), CD4 cell count (n=15), and HIV viral load (n=35) were estimated with multiple imputation models.

§ Adjusted model includes age, sex, pubertal stage, orphanhood, socioeconomic status, physical activity, calcium and vitamin D intake, age at ART initiation, years of TDF exposure, CD4 cell count, and viral load.

Older age (*vs* younger age), male sex (*vs* female sex), being an orphan (*vs* not an orphan), older age at ART initiation (*vs* younger age at initiation), and TDF exposure were all associated with lower LS-BMAD Z-score in unadjusted analysis (table 4). A weak association was detected between low CD4 cell count and low LS-BMAD Z-score. Older age and male sex remained associated, and earlier Tanner stage (*vs* later Tanner stage) showed an association, with low LS-BMAD Z-score after adjustment for orphanhood, socioeconomic status, physical activity, calcium and vitamin D intake, ART initiation, TDF exposure, CD4 count, viral load as well as age, sex, and pubertal stage.

**Table 4:** Characteristics associated with LS-BMAD Z-score in participants with HIV

	n (total N=279)*	**Mean LS-BMAD Z-score**†	**Crude β coefficient (95%CI)**‡	**p value**	**Adjusted β coefficient (95% CI)**‡§	**p value**
**Age**						
8–10 years	94	−0·11	1 (ref)	..	1 (ref)	..
11–13 years	94	−0·31	−0·24 (−0·62 to 0·13)	..	−0·36 (−0·86 to 0·15)	..
14–16 years	91	−1·13	−0·99 (−1·34 to −0·60)	<0·0001	−1·26 (−1·96 to −0·55)	0·0012
**Sex**						
Male	142	−0·89	1 (ref)	..	1 (ref)	..
Female	137	−0·12	0·76 (0·43 to 1·08)	<0·0001	0·77 (0·45 to 1·09)	<0·0001
**Pubertal stage**						
Tanner 1	117	−0·37	1 (ref)	..	1 (ref)	..
Tanner 2	59	−0·65	−0·30 (−0·75 to 0·15)	..	0·38 (−0·12 to 0·88)	..
Tanner 3	55	−0·51	−0·16 (−0·62 to 0·30)	..	0·56 (−0·01 to 1·13)	..
Tanner 4	44	−0·70	−0·32 (−0·89 to 0·25)	..	1·05 (0·32 to 1·78)	..
Tanner 5	12	−0·56	−0·29 (−1·14 to 0·57)	0·67	1·31 (0·36 to 2·25)	0·029
**Socioeconomic status**						
High: tertile 3	78	−0·48	1 (ref)	..	1 (ref)	..
Middle: tertile 2 (middle)	93	−0·49	−0·02 (−0·43 to 0·40)	..	0·02 (−0·36 to 0·41)	..
Low: tertile 1	108	−0·56	−0·09 (−0·51 to 0·32)	0·89	−0·14 (−0·55 to 0·26)	0·65
**Orphanhood**						
Not an orphan	154	−0·32	1 (ref)	..	1 (ref)	..
One or both parents dead	113	−0·77	−0·44 (−0·77 to −0·11)	0·010	−0·25 (−0·59 to 0·09)	0·14
**Physical activity level**						
High: >3000 MET minutes per week	76	−0·38	1 (ref)	..	1 (ref)	..
Moderate: 600–3000 MET minutes per week	69	−0·60	−0·23 (−0·68 to 0·22)	..	−0·15 (−0·58 to 0·28)	..
Low: <600 MET minutes per week	134	−0·55	−0·15 (−0·54 to 0·25)	0·39	−0·11 (−0·50 to 0·28)	0·77
**Daily dietary calcium intake**						
Moderate: 300–450 mg/day	95	−0·38	1 (ref)	..	1 (ref)	..
Low: 150–299 mg/day	60	−0·46	−0·07 (−0·53 to 0·39)	..	−0·05 (−0·50 to 0·41)	..
Very low: <150 mg/day	124	−0·64	−0·23 (−0·59 to 0·14)	0·46	−0·16 (−0·51 to 0·20)	0·69
**Daily dietary vitamin D intake**						
Moderate: 6·0–7·9 μg/day	50	−0·22	1 (ref)	..	1 (ref)	..
Low: 4·0–5·9 μg/day	190	−0·56	−0·36 (−0·95 to 0·23)	..	−0·28 (−0·74 to 0·18)	..
Very low: <4·0 μg/day	39	−0·64	−0·27 (−0·73 to 0·19)	0·40	−0·20 (−0·79 to 0·39)	0·42
**CD4 count**						
>500 cells per μL	213	−0·43	1 (ref)	..	1 (ref)	..
<500 cells per μL	54	−0·82	−0·39 (−0·80 to 0·02)	0·060	−0·06 (−0·48 to 0·36)	0·78
**HIV viral load**						
>1000 RNA copies per mL	54	−0·71	1 (ref)	..	1 (ref)	..
<1000 RNA copies per mL	207	−0·45	0·26 (−0·15 to 0·68)	0·21	0·07 (−0·34 to 0·48)	0·75
**Age at ART initiation**						
<4 years	148	−0·18	1 (ref)	..	1 (ref)	..
4–8 years	83	−0·79	−0·61 (−0·99 to −0·24)	..	−0·28 (−0·69 to 0·13)	..
>8 years	48	−1·06	−0·83 (−1·27 to −0·38)	<0·0001	−0·36 (−0·86 to 0·15)	0·29
**TDF years of exposure**						
None	184	−0·26	1 (ref)	..	1 (ref)	..
<4 years	59	−0·97	−0·70 (−1·11 to −0·29)	..	−0·38 (−0·81 to 0·05)	..
≥4 years	36	−1·04	−0·72 (−1·25 to −0·19)	<0·0001	−0·19 (−0·75 to 0·38)	0·25

LS-BMAD=lumbar spine bone mineral apparent density. MET=resting metabolic rate. ART=antiretroviral therapy. TDF=tenofovir disoproxil fumarate.

* Non-missing data.

† Arithmetic mean of non-missing data.

‡ β coefficient from linear regression indicates the Z-score difference from the reference category; lower values indicate lower Z-scores; missing data for total-body less-head bone mineral content for lean mass adjusted for height Z-score (n=24), LS-BMAD Z-score (n=24), pubertal stage (n=16), orphanhood (n=13), CD4 cell count (n=15), and HIV viral load (n=35) were estimated with multiple imputation models.

§ Adjusted model includes age, sex, pubertal stage, orphanhood, socioeconomic status, physical activity, calcium and vitamin D intake, age at ART initiation, years of TDF exposure, CD4 cell count, and viral load.

Current TDF use was associated with a 0·41 (95% CI 0·08–0·74; p=0·015) lower TBLH-BMC^LBM^ Z-score and 0·31 (0·08–0·69; p=0·12) lower LS-BMAD Z-score, compared with Z-scores in children with HIV on non-TDF-based ART, after adjustment for age, sex, pubertal stage, orphanhood, socioeconomic status, physical activity, calcium and vitamin D intake, CD4 cell count, HIV viral load, and age at ART initiation.

## Discussion

The main finding of this study was that despite ART, marked deficits in size-adjusted bone density are common among children with HIV, who have a substantially higher prevalence of low bone density (a deficit of 2 SDs or more from reference mean values) than their peers without HIV. The negative associations between HIV and size-adjusted bone density were more pronounced with pubertal maturation. Orphanhood and use of tenofovir were associated with the bone deficits in peripubertal children with HIV.

Across all age groups, children with HIV showed delayed pubertal stage compared with their peers without HIV. Despite low numbers, the association between HIV and size-adjusted bone density was most marked towards the end of puberty. This finding suggests cumulatively lower bone accrual throughout puberty in children with HIV, relative to children without HIV. Importantly, if bone accrual remains compromised, children with HIV will reach substantially lower PBM in adulthood. Girls with HIV had substantially lower TBLH-BMC^LBM^ Z-scores than girls without HIV, which became most apparent in later puberty; this level of deficit was not seen in boys. Both male and female children with HIV had lower LS-BMAD Z-scores and a greater risk of low LS-BMAD (ie, Z-score less than −2) than those without HIV. Boys with HIV in early puberty were at the greatest risk of low LS-BMAD. These results highlight the need for interventions to optimise bone density before skeletal growth is completed. A number of interventions might be beneficial. In Zimbabwe and Zambia, a trial is underway to assess whether supplementation of vitamin D_3_ and calcium ameliorates bone mineralisation deficits in adolescents growing up with HIV (Pan African Clinical Trials Registry number PACTR202009897660297). Furthermore, our study identified lower physical activity in children with HIV than in their uninfected peers. High impact physical activity is thought to be osteogenic, and has been associated with improved bone density at the hip in healthy adolescents.^24^ In 2021, a small randomised trial in young adults living with HIV suggested that a combination of resistance and aerobic exercise might improve bone mass;^27^ whether the same is applicable for children with HIV remains to be confirmed. In 2019, the first small randomised controlled trial of the oral bisphosphonate, alendronate, given to children with HIV, showed some evidence of improved bone density over 48 weeks of treatment;^28^ however, longer term effects on PBM, fracture risk, and rare side-effects are unknown.

The deficits we identified in TBLH-BMC^LBM^ in girls at the end of puberty is a concern. Bone density is compromised during pregnancy and lactation when skeletal calcium reserves are mobilised,^29^ and even healthy adolescent mothers might have a compromised PBM.^30^ Given that in 2019, 24% of young women living in Zimbabwe had given birth before the age of 18 years,^31^ our findings have implications for the recovery of skeletal mineralisation post partum and subsequent adult fracture risk.^32^

Children with HIV had a higher prevalence of stunting than with uninfected children. Furthermore, a higher proportion of children with HIV who were stunted had lower lumbar spine bone density than stunted children without HIV. Although stunting increases the risk of poor skeletal growth, we have shown that stunting is not a sufficient clinical proxy for low size-adjusted bone density. Interestingly, in children with HIV, deficits in standing height were greater than in sitting height, suggesting that HIV infection might have a greater effect on appendicular (limb length) than axial (spinal length) skeletal growth. Appendicular growth occurs more rapidly than axial growth before puberty. Exposures directly before puberty might preferentially affect appendicular development, whereas exposures during puberty might have a greater effect on axial development.^33^ Thus, our findings suggest that HIV and its treatment prepubertally could be particularly important in influencing linear growth.

Notably, ART drugs themselves might cause accelerated bone loss. We observed a strong and consistent association between TDF exposure and bone deficits, particularly affecting TBLH-BMC^LBM^. Children exposed to TDF for 4 or more years had, on average, a 0·52 SD deficit in TBLH-BMC^LBM^ Z-score compared with children with HIV who had not received TDF. This finding represents a clinically important effect size, as a 0·5 SD reduction in bone density increases fracture risk by 50%, both in children and, if sustained, adults.^34^ Although bone loss following TDF initiation in adults is well recognised, studies of TDF effects on bone outcomes in children have been inconsistent. For example, among 74 Brazilian adolescents (mean age 17·3 years [SD 1·8]), increased duration of TDF use was associated with lower (albeit non size-adjusted) lumbar spine and total body bone density;^35^ whereas a larger study of 394 Thai adolescents of a similar age (median 16·1 years [IQR 14·7–17·4) found no such association for (non-size adjusted) total body bone density or (size-adjusted) LS-BMAD.^36^

Our study supports the hypothesis that TDF use has a detrimental effect on bone health in children. Notably, we observed a dose response, with increased TDF exposure associated with pronounced bone deficits. TBLH-BMC^LBM^ largely represents cortical bone (whereas LS-BMAD reflects predominantly trabecular bone), and therefore our findings suggest TDF might particularly affect the mineralisation of cortical bone to reduce density, which is consistent with TDF-induced renal tubulopathy with phosphaturia leading to skeletal hypomineralisation.^37^ TDF is a well tolerated drug and remains one of the recommended component drugs of a first-line ART regimen, and is thus extensively used in sub-Saharan Africa, in both children and adults. In Zimbabwe, TDF is mostly available at an adult dose as part of a combination ART regimen, and use is recommended only in children weighing more than 25 kg or older than 10 years. However, due to limited availability of ART options, TDF is often prescribed in younger children or those of lower weight, effectively providing a higher than recommended dose. Our findings add to growing evidence supporting the replacement of TDF with tenofovir alafenamide, a prodrug of TDF that is associated with substantially fewer effects on bone and kidneys.^38^ This issue is of particular importance in adolescence, a period of rapid bone accrual, because reaching PBM might otherwise be compromised by TDF use.

The mechanism by which HIV compromises musculoskeletal development is likely to be multifactorial. HIV promotes dysregulated systemic immune activation, which is not completely reversed by ART.^39^ During childhood, bone formation usually predominates over bone resorption, but the proinflammatory milieu leads to an imbalance in osteoblastic and osteoclastic activity, promoting increased bone resorption relative to formation. Other factors that can compromise skeletal development include inadequate dietary calcium, vitamin D deficiency,^25^ and low levels of physical activity.^24^ Although in this study, children with HIV reported lower physical activity than uninfected children, low intake of both calcium and vitamin D were common regardless of HIV status. Orphanhood, more common in children with HIV, was independently associated with low TBLH-BMC^LBM^. Orphanhood might be a proxy for social and emotional deprivation, which, along with biological and nutritional factors, could disrupt growth.

The strengths of the study were being well powered, the inclusion of an HIV-uninfected comparison group from broadly the same socioeconomic background, and collection of detailed data on risk factors, including physical activity and vitamin D and calcium intake, via a standardised tool adapted for the local context. ISCD-recommended size-adjustment methods were used for DXA data, which were particularly crucial given height differences (levels of stunting) in the population studied.^16^, ^18^ In addition, sex-matched and age-matched Z-scores were used for size-adjusted bone density measures, which have greater clinical interpretability than absolute measures.

We acknowledge several limitations. Data were cross-sectional and therefore causality cannot be inferred. In the absence of local or African reference population data, the reference data used to generate the Z-scores for TBLH-BMC^LBM^ and LS-BMAD were obtained from a paediatric reference population in the UK, with use of the same DXA manufacturer and software version to derive Z-scores (as recommended by the ISCD).^16^ This reference population might not be comparable to the children in this study. Although the study population Z-score SDs for TBLH-BMC^LBM^ were close to 1, those for LS-BMAD were further from 1, suggesting the UK reference population might be a better fit for total body data than lumbar spine data, or that the size-adjustment should be further validated in African populations. DXA is unable to distinguish between bone density deficits due to hypomineralisation versus other architectural properties of bone. Only current CD4 cell count and HIV viral load were available as markers of HIV disease severity; annual trends in these measures would be more appropriate as growth occurs over a long period. Only 5 (2%) children with HIV reported non-perinatal infection and their inclusion is highly unlikely to have biased results. Aside from examining TDF versus non-TDF ART regimens, we did not have sufficient power to further categorise the many ART regimens and examine associations with other individual treatments. Furthermore, although we examined three-way interactions between age, puberty, and HIV on size-adjusted bone density, the study was not powered to detect the true differences between these strata. Although we aimed to collect comprehensive data on factors associated with size-adjusted bone density and growth, residual confounding might have been introduced by other past or current biological or environmental factors. Due to delays in engineering maintenance, DXA measurements were missing for a minority of children, in addition to a small amount of missing covariate data. We used multiple imputation with chained equations to impute these data, which were subject to a missing at random assumption. Our findings are therefore valid under this assumption and the specified imputation model. Finally, reaching PBM is estimated to occur in people in their early 20s, and therefore bone accrual might continue to consolidate after the age range examined.

To our knowledge, this study is the largest to investigate the relationship between HIV infection and skeletal health in children in sub-Saharan Africa in the ART era. Although ART results in immune reconstitution and has dramatically improved survival, this study highlights the importance of addressing the long-term adverse effects of HIV infection on musculoskeletal health in populations of children, particularly in sub-Saharan Africa. HIV programmes need to focus beyond delivery of ART, to develop strategies for prevention and management of the long-term effects of HIV infection, including on musculoskeletal health, to ensure optimum health in children as they enter adulthood. Longitudinal studies with follow-up of children during and after puberty are required to understand the extent of catch-up growth and bone accrual. Indeed, the population reported have now completed the 12-month follow-up in the IMVASK study and results are expected soon.

## Data sharing

Anonymised research data will be made available for sharing on the London School of Hygiene & Tropical Medicine (LSHTM) open access data repository (LSHTM Data Compass).

## Declaration of interests

RR (grant number 206764/Z/17/Z) and RAF (grant number 206316/Z/17/Z) are funded by the Wellcome Trust. CM-K is funded by a National Institute of Health Fogarty Trent Fellowship. AMR is partially supported by the UK Medical Research Council (MRC) and the UK Department for International Development (DFID) under the MRC/DFID Concordat agreement, which is also part of the European and Developing Countries Clinical Trials Partnership 2 programme supported by the EU (grant number MR/R010161/1). All other authors declare no competing interests.

## References

[1] Williams PL, Jesson J (2018). Growth and pubertal development in HIV-infected adolescents. Curr Opin HIV AIDS.

[2] Kekitiinwa A, Lee KJ, Walker AS (2008). Differences in factors associated with initial growth, CD4, and viral load responses to ART in HIV-infected children in Kampala, Uganda, and the United Kingdom/Ireland. J Acquir Immune Defic Syndr.

[3] Hernandez CJ, Beaupré GS, Carter DR (2003). A theoretical analysis of the relative influences of peak BMD, age-related bone loss and menopause on the development of osteoporosis. Osteoporos Int.

[4] Lowenthal ED, Bakeera-Kitaka S, Marukutira T, Chapman J, Goldrath K, Ferrand RA (2014). Perinatally acquired HIV infection in adolescents from sub-Saharan Africa: a review of emerging challenges. Lancet Infect Dis.

[5] Szubert AJ, Musiime V, Bwakura-Dangarembizi M (2015). Pubertal development in HIV-infected African children on first-line antiretroviral therapy. AIDS.

[6] Slogrove AL, Schomaker M, Davies MA (2018). The epidemiology of adolescents living with perinatally acquired HIV: a cross-region global cohort analysis. PLoS Med.

[7] Arpadi SM, Shiau S, Strehlau R (2016). Efavirenz is associated with higher bone mass in South African children with HIV. AIDS.

[8] Shiau S, Yin MT, Strehlau R (2020). Deficits in bone architecture and strength in children living with HIV on antiretroviral therapy. J Acquir Immune Defic Syndr.

[9] Arpadi SM, Shiau S, Marx-Arpadi C, Yin MT (2014). Bone health in HIV-infected children, adolescents and young adults: a systematic review. J AIDS Clin Res.

[10] Crabtree N, Ward K, Allgrove J, Shaw NJ (2015). Bone densitometry: current status and future perspective. Calcium and bone disorders in children and adolescents.

[11] Rukuni R, Gregson C, Kahari C (2020). The impact of vertical HIV infection on child and adolescent skeletal development in Harare, Zimbabwe (IMVASK Study): a protocol for a prospective cohort study. BMJ Open.

[12] Aurpibul L, Cressey TR, Sricharoenchai S (2015). Efficacy, safety and pharmacokinetics of tenofovir disoproxil fumarate in virologic-suppressed HIV-infected children using weight-band dosing. Pediatr Infect Dis J.

[13] Filteau S, Rehman AM, Yousafzai A (2016). Associations of vitamin D status, bone health and anthropometry, with gross motor development and performance of school-aged Indian children who were born at term with low birth weight. BMJ Open.

[14] Food and Nutrition Technical Assistance Project (August, 2006). Developing and validating simple indicators of dietary quality and energy intake of infants and young children in developing countries: summary of findings from analysis of 10 data sets. https://www.fantaproject.org/sites/default/files/resources/IYCF_Datasets_Summary_2006.pdf.

[15] Hologic (August, 2015). Apex data archiving best practises. https://www.hologic.com/sites/default/files/2018-05/APEX%20Data%20Archiving%20Best%20Practices%20Guide%20MAN-03906%20English%20Rev%20005%2008_13.pdf.

[16] Crabtree NJ, Arabi A, Bachrach LK (2014). Dual-energy X-ray absorptiometry interpretation and reporting in children and adolescents: the revised 2013 ISCD Pediatric Official Positions. J Clin Densitom.

[17] Carter DR, Bouxsein ML, Marcus R (1992). New approaches for interpreting projected bone densitometry data. J Bone Miner Res.

[18] Crabtree NJ, Shaw NJ, Bishop NJ (2017). Amalgamated reference data for size-adjusted bone densitometry measurements in 3598 children and young adults—the ALPHABET Study. J Bone Miner Res.

[19] Palchetti CZ, Szejnfeld VL, de Menezes Succi RC (2015). Impaired bone mineral accrual in prepubertal HIV-infected children: a cohort study. Braz J Infect Dis.

[20] DiMeglio LA, Wang J, Siberry GK (2013). Bone mineral density in children and adolescents with perinatal HIV infection. AIDS.

[21] Cole TJ, Freeman JV, Preece MA (1998). British 1990 growth reference centiles for weight, height, body mass index and head circumference fitted by maximum penalized likelihood. Stat Med.

[22] Szubert AJ, Musiime V, Bwakura-Dangarembizi M (2015). Pubertal development in HIV-infected African children on first-line antiretroviral therapy. AIDS.

[23] Fox AM (2012). The HIV-poverty thesis re-examined: poverty, wealth or inequality as a social determinant of HIV infection in sub-Saharan Africa?. J Biosoc Sci.

[24] Deere K, Sayers A, Rittweger J, Tobias JH (2012). Habitual levels of high, but not moderate or low, impact activity are positively related to hip BMD and geometry: results from a population-based study of adolescents. J Bone Miner Res.

[25] Penner J, Ferrand RA, Richards C, Ward KA, Burns JE, Gregson CL (2018). The impact of vitamin D supplementation on musculoskeletal health outcomes in children, adolescents, and young adults living with HIV: a systematic review. PLoS One.

[26] Gregson CL, Hartley A, Majonga E (2019). Older age at initiation of antiretroviral therapy predicts low bone mineral density in children with perinatally-infected HIV in Zimbabwe. Bone.

[27] Ghayomzadeh M, Earnest CP, Hackett D (2021). Combination of resistance and aerobic exercise for six months improves bone mass and physical function in HIV infected individuals: a randomized controlled trial. Scand J Med Sci Sports.

[28] Jacobson DL, Lindsey JC, Gordon C (2019). Alendronate improves bone mineral density in children and adolescents perinatally infected with human immunodeficiency virus with low bone mineral density for age. Clin Infect Dis.

[29] Winter EM, Ireland A, Butterfield NC (2020). Pregnancy and lactation, a challenge for the skeleton. Endocr Connect.

[30] Bezerra FF, Mendonça LM, Lobato EC, O'Brien KO, Donangelo CM (2004). Bone mass is recovered from lactation to postweaning in adolescent mothers with low calcium intakes. Am J Clin Nutr.

[31] UNICEF Data (2019). Early childbearing—percentage of women (aged 20–24 years) who gave birth before age 18. https://data.unicef.org/topic/child-health/adolescent-health/.

[32] Ward KA, Adams JE, Mughal MZ (2005). Bone status during adolescence, pregnancy and lactation. Curr Opin Obstet Gynecol.

[33] Bass S, Delmas PD, Pearce G, Hendrich E, Tabensky A, Seeman E (1999). The differing tempo of growth in bone size, mass, and density in girls is region-specific. J Clin Invest.

[34] Goulding A, Jones IE, Taylor RW, Manning PJ, Williams SM (2000). More broken bones: a 4-year double cohort study of young girls with and without distal forearm fractures. J Bone Miner Res.

[35] Schtscherbyna A, Pinheiro MF, Mendonça LM (2012). Factors associated with low bone mineral density in a Brazilian cohort of vertically HIV-infected adolescents. Int J Infect Dis.

[36] Sudjaritruk T, Bunupuradah T, Aurpibul L (2017). Impact of tenofovir disoproxil fumarate on bone metabolism and bone mass among perinatally HIV-infected Asian adolescents. Antivir Ther.

[37] Grant PM, Cotter AG (2016). Tenofovir and bone health. Curr Opin HIV AIDS.

[38] Ray AS, Fordyce MW, Hitchcock MJM (2016). Tenofovir alafenamide: a novel prodrug of tenofovir for the treatment of human immunodeficiency virus. Antiviral Res.

[39] Wilson EMP, Sereti I (2013). Immune restoration after antiretroviral therapy: the pitfalls of hasty or incomplete repairs. Immunol Rev.

