# Supplementary material for: Effect of HIV infection on growth and bone density in peripubertal children in the era of antiretroviral therapy: a cross-sectional study in Zimbabwe
Source: Lancet Child Adolesc Health. 2021 Aug;5(8):569–81. doi: 10.1016/S2352-4642(21)00133-4 (PMC8295041; doi:10.1016/S2352-4642(21)00133-4)
Supplement: Supplementary appendix [file mmc1.pdf]

THE LANCET
Child & Adolescent Health

Supplementary appendix

This appendix formed part of the original submission and has been peer reviewed. We post it as supplied by the authors.

Supplement to: Rukuni R, Rehman AM, Mukwasi-Kahari C, et al. Effect of HIV infection on growth and bone density in peripubertal children in the era of antiretroviral therapy: a cross-sectional study in Zimbabwe. *Lancet Child Adolesc Health* 2021; published online June 14. https://doi.org/10.1016/S2352-4642(21)00133-4.

Supplementary materials (Tables 1-5) for 'Effect of HIV infection on growth and bone density in peripubertal children in the era of antiretroviral therapy: a cross-sectional study in Zimbabwe'. Rukuni et al.

Supplementary Table 1. Characteristics of participants who did and did not have bone outcomes measured by DXA

		DXA (n=571)		Missing DXA (n=38)		p value
		N	n (%)[a]	N	n (%)[a]	
Socio-demographic factors	Age years, mean (SD)	571	12.4 (2.5)	38	12.9 (2.7)	0.269
	Female sex	571	287 (50.3)	38	19 (50.0)	0.975
	Socioeconomic status (SES)					
	Tertile 1 (low)	571	191 (33.5)	38	12 (31.6)	0.456
	Tertile 2 (middle)		187 (32.8)		16 (42.1)	
	Tertile 3 (high)		193 (33.8)		10 (26.3)	
	Orphanhood (one or both parents dead) [b]	556	132 (23.7)	37	11 (29.7)	0.410
Lifestyle factors	Outdoor time >2 hours/day	571	365 (64.0)	38	30 (79.0)	0.060
	Physical activity level					
	Low, <600 MET mins/week	571	244 (42.7)	38	18 (47.4)	0.464
	Moderate, 600-3000 MET mins/week		153 (26.8)		12 (31.6)	
	High, >3000 MET mins/week		174 (30.5)		8 (21.1)	
	Daily dietary calcium intake					
	Very low, <150 mg/day	571	253 (44.3)	38	18 (47.4)	0.459
	Low, 150-299 mg/day		123 (21.5)		5 (13.2)	
	Moderate, 300–450 mg/day		195 (34.2)		15 (39.5)	
	Daily dietary vitamin D					
	Very low, <4.0 mcg/day	571	74 (13.0)	38	3 (7.9)	0.393
	Low, 4.0-5.9 mcg/day		378 (66.2)		24 (63.2)	
	Moderate, 6.0-8.0 mcg/day		119 (20.8)		11 (29.0)	
Pubertal stage	Tanner I	553	175 (31.7)	37	11 (30.0)	0.407
	Tanner II		121 (21.9)		7 (18.9)	
	Tanner III		98 (17.7)		10 (27.0)	
	Tanner IV		127 (23.0)		9 (24.3)	
	Tanner V		32 (5.8)		0 (0.0)	
	Pubertal delay [c]	239	7 (2.9)	20	1 (5.0)	0.479
HIV characteristics	Age at HIV diagnosis years, median (IQR)	279	2.9 (1.2; 5.9)	24	3.5(1.5;5.5)	0.787
	Age at ART initiation years, median (IQR)	279	3.7 (1.9; 7.0)	24	4.0 (1.6;5.8)	0.759
	ART duration years, median (IQR)	279	8.0 (6.1; 9.6)	24	8.3 (7.3;9.4)	0.340
	% life on ART, mean (SD)	279	65.1 (22.5)	24	69.4 (17.0)	0.359
	Current TDF use	571	95 (16.6)	38	7 (18.4)	0.776
	Viral load <1,000 copies/ml	261	207 (79.3)	7	5 (71.4)	0.613
	CD4 count <500 cells/µL	267	54 (20.2)	21	4 (19.1)	0.897
Anthropometry	Standing height Z-score, mean (SD)	571	-1.1 (1.3)	37	-1.6 (1.5)	0.044
	Height-for-age Z-score <-2	571	112 (19.6)	37	9 (24.3)	0.487
	Sitting height for age Z-score, mean (SD)	568	-1.73 (1.2)	38	-1.92 (1.3)	0.354
	Sitting height-for-age Z-score <-2	568	214 (37.7)	38	14 (36.8)	0.918
	Weight-for-age Z-score, mean (SD)	569	-0.98 (1.3)	38	-1.36 (1.2)	0.075
	Weight-for-age Z-score <-2	569	100 (17.6)	38	5 (13.2)	0.486
	BMI Z-score, mean (SD)	569	-0.44 (1.1)	37	-0.64 (1.2)	0.283
	BMI Z-score <-2	569	38 (6.7)	37	8 (21.6)	0.001
	Mid upper arm circumference	569	20.6 (3.2)	38	20.4 (3.0)	0.719

[a] *n(%) unless stated otherwise;* [b] *one or both parents dead;* [c] *definition included girls >13 years and boys >14 years*

Supplementary Table 2. Characteristics of participants with complete data for study outcome, tanner stage, orphanhood and among CWH with CD4 cell count and HIV viral load, compared to those with missing data for one or more variables

		Non-missing (n=512)		Missing (n=97)		p value
		N	n (%)[a]	N	n (%)[a]	
Socio-demographic factors	Age years, mean (SD)	512	12.4 (2.5)	97	12.8 (2.5)	0.230
	Female sex	512	256 (50.0)	97	50 (51.6)	0.780
	Socioeconomic status (SES)					
	Tertile 1 (low)	512	173 (33.8)	97	30 (30.9)	0.182
	Tertile 2 (middle)		163 (31.8)		40 (41.2)	
	Tertile 3 (high)		176 (34.4)		27 (27.8)	
	Orphanhood (one or both parents dead) [b]	512	115 (22.5)	81	28 (34.6)	0.018
Lifestyle factors	Outdoor time >2 hours/day	512	320 (62.5)	97	75 (77.3)	0.005
	Physical activity level					
	Low, <600 MET mins/week	512	211 (41.2)	97	51 (52.6)	0.038
	Moderate, 600-3000 MET mins/week		138 (27.0)		27 (27.8)	
	High, >3000 MET mins/week		163 (31.8)		19 (19.6)	
	Daily dietary calcium intake					
	Very low, <150 mg/day	512	227 (44.3)	97	44 (45.4)	0.451
	Low, 150-299 mg/day		112 (21.9)		16 (16.5)	
	Moderate, 300–450 mg/day		173 (33.8)		37 (38.1)	
	Daily dietary vitamin D					
	Very low, <4.0 mcg/day	512	66 (12.9)	97	11 (11.3)	0.882
	Low, 4.0-5.9 mcg/day		338 (66.0)		64 (66.0)	
	Moderate, 6.0-8.0 mcg/day		108 (21.1)		22 (22.7)	
Pubertal stage	Tanner I	512	163 (31.8)	78	23 (29.5)	0.294
	Tanner II		108 (21.1)		20 (25.6)	
	Tanner III		89 (17.4)		19 (24.4)	
	Tanner IV		122 (23.8)		14 (18.0)	
	Tanner V		30 (5.9)		2 (2.6)	
	Pubertal delay [c]	222	7 (3.2)	37	1 (2.7)	0.680
HIV characteristics	Age at HIV diagnosis years, median (IQR)	226	3.0 (1.1; 5.8)	77	3.0 (1.5;5.7)	0.787
	Age at ART initiation years, median (IQR)	226	3.7 (1.8; 7.0)	77	3.6 (1.8;6.4)	0.922
	ART duration years, median (IQR)	226	7.9 (6.0; 9.4)	77	8.6 (6.5;9.7)	0.094
	% life on ART, mean (SD)	226	64.9 (22.6)	77	67.0 (20.7)	0.481
	Current TDF use	226	77 (34.1)	77	25 (32.5)	0.797
	Viral load <1,000 copies/ml	226	179 (79.2)	42	33 (78.6)	0.926
	CD4 count <500 cells/µL	226	44 (19.5)	62	14 (22.6)	0.588
Anthropometry	Standing height Z-score, mean (SD)	512	-1.1 (1.2)	96	-1.6 (1.3)	0.0001
	Height-for-age Z-score <-2	512	95 (18.6)	96	26 (27.1)	0.055
	Sitting height for age Z-score, mean (SD)	511	-1.7 (1.2)	95	-2.1 (1.2)	0.003
	Sitting height-for-age Z-score <-2	511	185 (36.2)	95	43 (45.3)	0.094
	Weight-for-age Z-score, mean (SD)	512	-0.9 (1.3)	95	-1.4 (1.3)	0.003
	Weight-for-age Z-score <-2	512	88 (17.2)	95	17 (17.9)	0.867
	BMI Z-score, mean (SD)	512	-0.4 (1.1)	94	-0.6 (1.2)	0.229
	BMI Z-score <-2	512	31 (6.1)	94	15 (16.0)	0.001
	Mid upper arm circumference	512	20.6 (3.2)	95	20.5 (2.9)	0.813

Supplementary Table 3. Number and percentage of participants by HIV status, pubertal stage, age group and sex

Tanner stage	Participants with HIV n=287 [a]				Participants without HIV n=303 [b]			
MALES (n=292)	Age (Years)				Age (Years)			
	8-10	11-13	14-16	Total	8-10	11-13	14-16	Total
Tanner I	41 (80.4)	12 (26.1)	4 (8.9)	57	39 (79.6)	6 (12.0)	0 (0.0)	45
Tanner II	10 (19.6)	19 (41.3)	10 (22.2)	39	9 (18.4)	25 (50.0)	0 (0.0)	34
Tanner III	0 (0.0)	13 (28.3)	9 (20.0)	22	1 (2.0)	13 (26.0)	10 (19.6)	24
Tanner IV	0 (0.0)	2 (4.3)	17 (37.8)	19	0 (0.0)	6 (12.0)	37 (72.5)	43
Tanner V	0 (0.0)	0 (0.0)	5 (11.1)	5	0 (0.0)	0 (0.0)	4 (7.8)	4
FEMALES (n=298)	Age (Years)				Age (Years)			
	8-10	11-13	14-16	Total	8-10	11-13	14-16	Total
Tanner I	46 (93.9)	12 (25.5)	2 (4.1)	60	24 (52.2)	0 (0.0)	0 (0.0)	24
Tanner II	3 (6.1)	12 (25.5)	5 (10.2)	20	17 (36.9)	18 (35.3)	0 (0.0)	35
Tanner III	0 (0.0)	20 (42.6)	13 (26.5)	33	5 (10.9)	23 (45.1)	1 (1.8)	29
Tanner IV	0 (0.0)	3 (6.4)	22 (44.9)	25	0 (0.0)	9 (17.6)	40 (71.4)	49
Tanner V	0 (0.0)	0 (0.0)	7 (14.3)	7	0 (0.0)	1 (2.0)	15 (26.8)	16

[a] *Tanner stage missing in 16 (of 303) children with HIV*
[b] *Tanner stage missing in 3 (of 306) children without HIV*

Supplementary Table 4. The Relationship Between Stunting (height-for-age Z-score<-2) and Low Size-adjusted Total Body and Lumbar Spine Bone Density (Z-score<-2) in the 279 children with HIV and 293 children without HIV who had DXA scans performed

**Participants with HIV (n=279)**	**TBLH-BMC^{LBM} Z-score <-2 n=29**	**TBLH-BMC^{LBM} Z-score ≥-2 n=250**	**p value**	**LS-BMAD Z-score <-2 n=40**	**LS-BMAD Z-score ≥-2 n=239**	**p value**
	**N (%)**			**N (%)**		
**Height-for-age Z-score <-2 n=88**	8 (27.6)	80 (32.0)	0.628	23 (57.5)	65 (27.2)	<0.001
**Height-for-age Z-score ≥-2 n=191**	21 (72.4)	170 (68.0)		17 (42.5)	174 (72.8)	
**Participants without HIV (n=293)**	**TBLH-BMC^{LBM} Z-score <-2 n=18**	**TBLH-BMC^{LBM} Z-score ≥-2 n=274**	**p value**	**LS-BMAD Z-score <-2 n=17**	**LS-BMAD Z-score ≥-2 n=276**	**p value**
	**N (%)**			**N (%)**		
**Height-for-age Z-score <-2 n=24**	1 (5.6)	23 (8.4)	0.488	2 (11.8)	22 (8.0)	0.478
**Height-for-age Z-score ≥-2 n= 269**	17 (94.4)	251 (91.6)		15 (88.2)	254 (92.0)	

Supplementary Table 5. Characteristics associated with TBLH-BMC^{LBM} and LS-BMAD Z-Score in participants without HIV

Characteristic	N=293[1]	TBLH-BMC^{LBM} Z-Score					LS-BMAD Z-Score				
		Mean TBLH-BMC^{LBM} Z-score[2]	Crude beta coefficient (95% CI)[3]	p value	Adjusted beta coefficient[a] (95% CI)[3,4]	p value	Mean LS BMAD Z-score[2]	Crude beta coefficient (95% CI)[3]	p value	Adjusted beta coefficient[a] (95% CI)[3,4]	p value
**Age (years)**											
8-10	96	-0.367	Ref	0.051	Ref	0.673	-0.066	Ref	0.159	Ref	0.001
11-13	94	-0.611	-0.24 (-0.53, 0.05)		-0.16 (-0.55, 0.23)		-0.280	-0.21 (-0.56, 0.13)		-0.56 (-1.02, -0.11)	
14-16	103	-0.254	0.10 (-0.18, 0.39)		-0.26 (-0.85, 0.34)		-0.372	-0.33 (-0.66, 0.01)		-1.31 (-1.97, -0.65)	
**Sex**											
Male	143	-0.562	Ref	0.012	Ref	0.047	-0.608	Ref	<0.001	Ref	<0.001
Female	150	-0.258	0.29 (0.07, 0.52)		0.24 (0.003, 0.49)		0.106	0.70 (0.43, 0.97)		0.53 (0.25, 0.81)	
**Pubertal status**											
Tanner I	68	-0.309	Ref	0.004	Ref	0.045	-0.253	Ref	0.034	Ref	0.001
Tanner II	65	-0.705	-0.41 (-0.74, -0.07)		-0.32 (-0.72, 0.09)		-0.291	-0.02 (-0.43, 0.38)		0.18 (-0.28, 0.65)	
Tanner III	49	-0.598	-0.26 (-0.63, 0.10)		-0.17 (-0.66, 0.33)		-0.287	-0.05 (-0.49, 0.40)		0.46 (-0.12, 1.04)	
Tanner IV	88	-0.283	0.004 (-0.31, 0.32)		0.21 (-0.40, 0.83)		-0.363	-0.12 (-0.50, 0.27)		0.93 (0.23, 1.63)	
Tanner V	20	0.170	0.48 (-0.01, 0.97)		0.63 (-0.14, 1.40)		-0.600	0.85 (0.26, 1.45)		1.86 (0.98, 2.74)	
**Socioeconomic status**											
SES Tertile 3 (high)	115	-0.387	Ref	0.660	Ref	0.615	-0.238	Ref	0.999	Ref	0.956
SES Tertile 2 (middle)	95	-0.477	-0.10 (-0.37, 0.18)		-0.14 (-0.42, 0.14)		-0.257	-0.007 (-0.34, 0.32)		-0.05 (-0.37, 0.27)	
SES Tertile 1 (low)	83	-0.353	0.03 (-0.26, 0.32)		-0.07 (-0.38, 0.21)		-0.231	0.001 (-0.35, 0.35)		-0.01 (-0.35, 0.33)	
**Orphanhood**											
Not an orphan	271	-0.420	Ref	0.246	Ref	0.205	-0.224	Ref	0.714	Ref	0.826
1 or 2 parents' dead	19	-0.161	0.28 (-0.19, 0.75)		0.30 (-0.17, 0.78)		-0.346	-0.10 (-0.67, 0.46)		-0.06 (-0.60, 0.48)	
**Physical activity**											
High, >3000 MET/wk	98	-0.288	Ref	0.357	Ref	0.249	-0.159	Ref	0.561	Ref	0.686
Mod, 600-3000 MET/wk	84	-0.488	-0.20 (-0.49, 0.10)		-0.23 (-0.53, 0.06)		-0.358	-0.19 (-0.55, 0.17)		-0.15 (-0.50, 0.20)	
Low, <600 MET/wk	111	-0.448	-0.16 (-0.43, 0.11)		-0.18 (-0.46, 0.09)		-0.228	-0.06 (-0.39, 0.28)		-0.09 (-0.41, 0.23)	
**Daily calcium intake**											
Mod, 300–450 mg/day	100	-0.413	Ref	0.996	Ref	0.805	-0.265	Ref	0.715	Ref	0.745
Low, 150-299 mg/day	64	-0.390	-0.01 (-0.33, 0.31)		-0.09 (-0.41, 0.23)		-0.149	0.13 (-0.25, 0.51)		0.07 (-0.30, 0.43)	
Very low, <150 mg/day	129	-0.409	0.01 (-0.26, 0.27)		-0.08 (-0.35, 0.19)		-0.271	-0.02 (-0.33, 0.30)		-0.07 (-0.37, 0.24)	
**Daily vitamin D intake**											
Mod, 6.0-7.9 mcg/day	69	-0.534	Ref	0.089	Ref	0.083	-0.210	Ref	0.761	Ref	0.815
Low, 4.0-5.9 mcg/day	189	-0.418	0.114 (-0.168, 0.397)		0.16 (-0.13, 0.44)		-0.230	-0.03 (-0.37, 0.31)		-0.005 (-0.33, 0.32)	
Very low, <4.0 mcg/day	35	-0.089	0.459 (0.056, 0.862)		0.48 (0.07, 0.89)		-0.372	-0.18 (-0.67, 0.31)		-0.14 (-0.62, 0.34)	

[1]Non-missing outcome data. [2]Arithmetic mean of non-missing outcome data. [3]Linear regression with robust standard errors. Missing data for TBLH-BMCLBM Z-score (n=14), LS BMAD Z-score (n=13), pubertal stage (n=3) and orphanhood (n=3) were imputed using chained equations. [4]Adjusted for age, sex, pubertal stage, orphanhood, socioeconomic status, physical activity, calcium and vitamin D intake
